# Cost of care for Alzheimer’s disease and related dementias in the United States: 2016 to 2060

**DOI:** 10.1038/s41514-024-00136-6

**Published:** 2024-02-08

**Authors:** Arindam Nandi, Nathaniel Counts, Janina Bröker, Sabrina Malik, Simiao Chen, Rachael Han, Jessica Klusty, Benjamin Seligman, Daniel Tortorice, Daniel Vigo, David E. Bloom

**Affiliations:** 1 The Population Council, 1 Dag Hammarskjold Plaza, New York, NY 10017 USA; 2 One Health Trust, Washington, DC USA; 3 Office of the Commissioner of Health & Mental Hygiene for the City of New York, New York, NY USA; 4 The Brookings Institution, Washington, DC USA; 5 Data for Decisions, LLC, Waltham, MA USA; 6 University of Heidelberg, Heidelberg, Germany; 7 Department of Molecular and Cellular Biology and The Center for Brain Science, Harvard University, Cambridge, MA USA; 8 Division of Geriatric Medicine, Department of Medicine, David Geffen School of Medicine, University of California, Los Angeles, CA USA; 9 Geriatrics Research, Education, and Clinical Center, Greater Los Angeles VA Health Care System, Los Angeles, CA USA; 10 College of the Holy Cross, Worcester, MA USA; 11 University of British Columbia, Vancouver, BC V6T 1Z4 Canada; 12 Harvard T.H. Chan School of Public Health, Boston, MA USA

**Keywords:** Health policy, Health care economics

## Abstract

Medical and long-term care for Alzheimer’s disease and related dementias (ADRDs) can impose a large economic burden on individuals and societies. We estimated the per capita cost of ADRDs care in the in the United States in 2016 and projected future aggregate care costs during 2020–2060. Based on a previously published methodology, we used U.S. Health and Retirement Survey (2010–2016) longitudinal data to estimate formal and informal care costs. In 2016, the estimated per patient cost of formal care was $28,078 (95% confidence interval [CI]: $25,893–$30,433), and informal care cost valued in terms of replacement cost and forgone wages was $36,667 ($34,025–$39,473) and $15,792 ($12,980–$18,713), respectively. Aggregate formal care cost and formal plus informal care cost using replacement cost and forgone wage methods were $196 billion (95% uncertainty range [UR]: $179–$213 billion), $450 billion ($424–$478 billion), and $305 billion ($278–$333 billion), respectively, in 2020. These were projected to increase to $1.4 trillion ($837 billion–$2.2 trillion), $3.3 trillion ($1.9–$5.1 trillion), and $2.2 trillion ($1.3–$3.5 trillion), respectively, in 2060.

## Introduction

The population of those aged 65 years and higher in the United States is projected to grow from 55 million in 2020 to 94 million in 2060^1^. With population aging, the burden of Alzheimer’s disease and related dementias (ADRDs) will also grow substantially. ADRDs represent a related set of conditions marked by progressive neurodegeneration, including Alzheimer’s disease, vascular dementia, dementia with Lewy bodies, and frontotemporal dementia. An estimated 6.5 million older Americans—almost three quarters of whom are older than 75—lived with Alzheimer’s dementia in 2022^2^. By 2060, this figure is projected to grow to 13.8 million people^2,3^. ADRDs can severely affect cognition, i.e., functions of the brain such as learning, memory, and reasoning; interfere with activities of daily living such as bathing, walking, or eating; and lead to neuropsychological dysfunctions, including anxiety, delirium, and psychosis, and eventual death^4,5^.

In 2013, Hurd et al. published a critical study estimating the cost of illness of dementia in the United States, which enabled the country to better understand the associated economic burden and support policy efforts to more effectively treat ADRDs^6^. Hurd et al. estimated that in 2010 the direct medical care and informal caregiving costs for dementia in the United States ranged between $157 billion and $215 billion, depending upon the methodology used for valuing informal care^6^. In 2015, Hurd et al. built on their study by forecasting potential future costs, helping the nation to understand how the magnitude of the threat may grow over time if left unaddressed^7^. If the prevalence rate of dementia remained at its 2010 level, the aggregate annual care cost is estimated to increase to $379–$511 billion by 2040, or to $1.5 trillion by 2050 (2010 US$)^7,8^.

Subsequently, other cost of illness studies proliferated. The Alzheimer’s Association, which publishes annual estimates of the direct medical care cost based on data from various sources, including the Medicare Current Beneficiary Survey and published studies of disease prevalence^2,9^, estimated that aggregate direct care cost would increase from $226 billion in 2015 to $1.1 trillion in 2050 (2015 US$)^10^. Further estimates abound, with substantial variations in cost components, study cohorts, analytical techniques, and findings^5,11–25^.

This study seeks to update and build on the analyses conducted by Hurd et al. to motivate a next wave of action on ADRDs in a rapidly aging country. National estimates of direct and indirect cost are based primarily on data from 2010 or earlier, and even smaller regional studies are based on data that are now a decade old^25,26^. Newer estimates are necessary for research and policymaking, especially considering the changed ADRDs care landscape due to the passage of the Affordable Care Act in 2010 and the National Alzheimer’s Project Act in 2011^27^. To the best of our knowledge, only one study has used national data up to 2016 to estimate the current out-of-pocket cost of dementia care^28^. Furthermore, future projection studies from Hurd et al. and others do not systematically quantify uncertainty and sometimes only report point estimates^7,8,10^. An accurate forecast of the future economic burden of ADRDs, projected based on the most recent data and adequately incorporating uncertainty, enables policymakers and stakeholders to invest appropriately in research and development for therapeutics and cost-effective care innovations to mitigate this potential economic toll on society^29–31^.

In this study, we projected the direct and indirect costs of ADRDs care in the United States in five-year intervals from 2020 to 2060, following the same methodology as Hurd et al., using the most recently available data and incorporating uncertainty. We used national longitudinal data from 2010–2016 to estimate the per capita care cost for ADRDs based on this previously published methodology^6^ and projected aggregate costs through 2060, accounting for uncertainty.

## Results

Table 1 presents the per patient cost of formal and informal ADRDs care in 2016 in 2020 US$. The estimated per patient cost of formal care was $28,078 (95% CI: $25,893–$30,433). The per patient cost of informal care valued in terms of replacement cost and forgone wages was $36,667 (95% CI: $34,025–$39,473) and $15,792 (95% CI: $12,980–$18,713), respectively. Total per patient costs including formal and informal care in terms of replacement cost and foregone wages were $64,745 (95% CI: $61,740–$67,909) and US$43,869 (95% CI: US$40,246–US$47,591), respectively.

**Table 1:** Per patient cost of ADRDs formal and informal care in the United States, 2016.

Spending type	Cost of ADRDs care in the United States (2020 US$)		
	Estimate	95% confidence interval	
Out-of-pocket:			
Home health care	185	108	271
Nursing home	7203	6155	8263
Total (A)	7943	6878	9078
Medicare spending:			
Home health care agencies	964	759	1191
Nursing home	1505	1395	1618
Total (B)	4365	3974	4794
Formal home care:			
Total	7220	5687	8917
Less: Medicare and OOP	1149	935	1381
Net total (C)	6071	4744	7554
Nursing home care:			
Total	18,406	17,057	19,791
Less: Medicare and OOP	8708	7587	9825
Net total (D)	9698	8453	10,912
Total care purchased in the marketplace (A + B + C + D)	28,078	25,893	30,433
Informal care: replacement cost (E)	36,667	34,025	39,473
Total care purchased in the marketplace plus caregiving time valued according to replacement cost (A + B + C + D + E)	64,745	61,740	67,909
Informal care: Foregone wage cost (F)	15,792	12,980	18,713
Total care purchased in the marketplace plus caregiving time valued at foregone wage cost (A + B + C + D + F)	43,869	40,246	47,591

Considering only those who reported non-zero costs, the estimated per patient cost of formal care in 2016 was $56,022 (95% CI: $43,136–$69,500). Total per patient costs including formal and informal care in terms of replacement cost and foregone wages were $92,689 (95% CI: $79,813–$106,240) and $71,813 (95% CI: $58,623–$85,188) respectively.

**Fig. 1:**
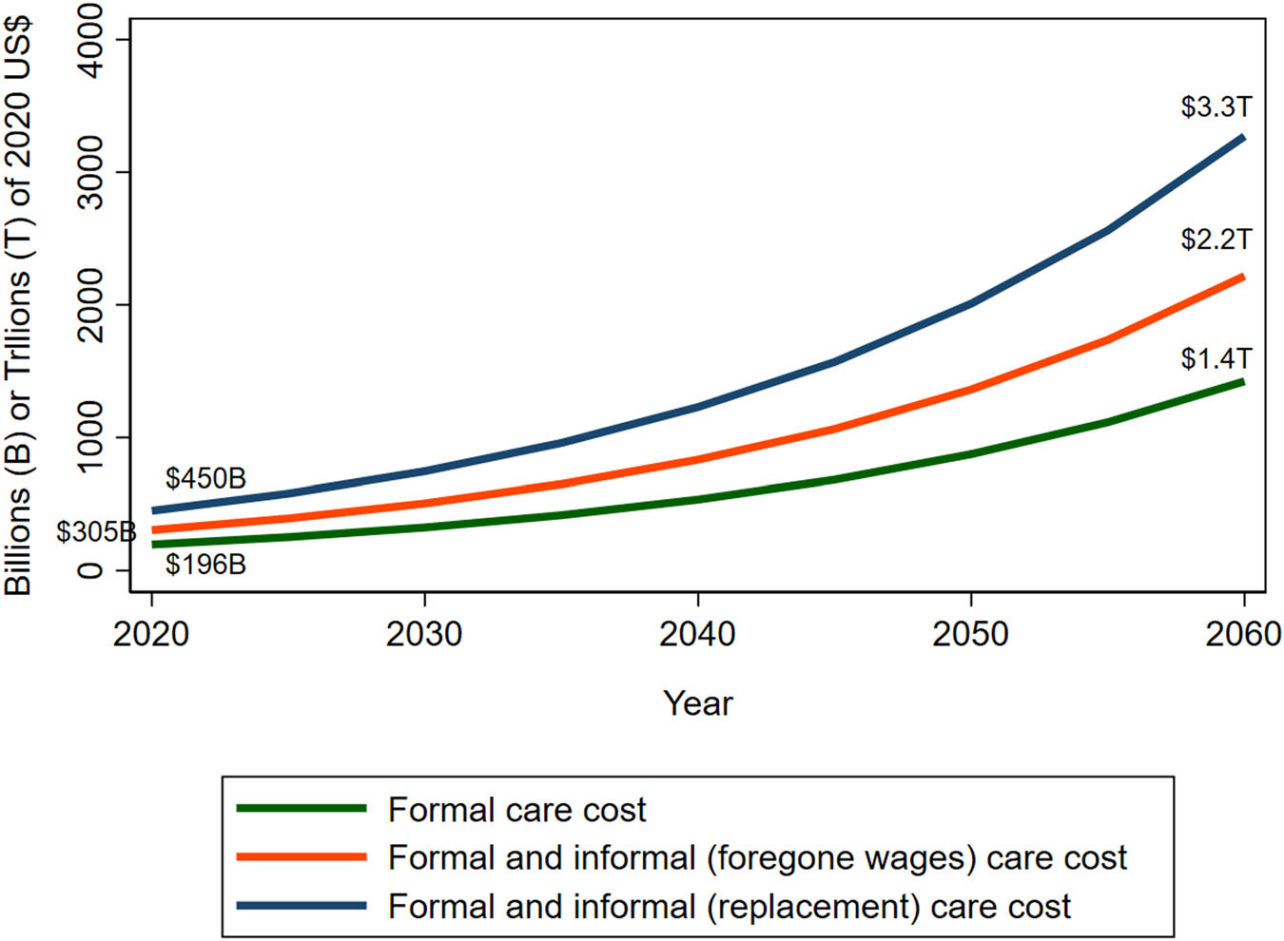
Projected cost of ADRDs formal and informal care in the United States, 2020 to 2060.

Figure 1 and Tables 2, 3, and 4 present the future projections of care cost (2020 US$). Under the base case scenario, the annual aggregate formal care cost for ADRDs was an estimated $196 billion (95% uncertainty range [UR]: $179–$213 billion) in 2020 and was projected to increase to $1.4 trillion (95% UR: $837 billion–$2.2 trillion) by 2060. Aggregate formal and informal care (replacement method) cost was estimated to increase from $450 billion (95% UR: $424–$478 billion) in 2020 to $3.3 trillion (95% UR: $1.9–$5.1 trillion) in 2060. Aggregate formal and informal care (foregone wages method) cost was estimated to increase from $305 billion (95% UR: $278–$333 billion) in 2020 to $2.2 trillion (95% UR: $1.3–$3.5 trillion) in 2060.

**Table 2:** Projected future cost of ADRDs formal and informal care in the United States.

Year	Total care purchased in the marketplace (billions of 2020 US$)	Total care purchased in the marketplace plus caregiving time valued according to replacement cost (billions of 2020 US$)	Total care purchased in the marketplace plus caregiving time valued at foregone wage costs (billions of 2020 US$)
Base case (3% annual cost inflation)			
2020	196 (179–213)	450 (424–478)	305 (278–333)
2025	252 (184–326)	580 (430–740)	393 (288–508)
2030	325 (233–430)	746 (542–979)	506 (363–671)
2035	417 (293–566)	959 (677–1292)	650 (456–878)
2040	535 (364–748)	1229 (842–1699)	833 (567–1160)
2045	683 (450–988)	1570 (1046–2247)	1064 (702–1532)
2050	875 (558–1295)	2010 (1293–2945)	1362 (867–2009)
2055	1115 (677–1714)	2562 (1571–3911)	1736 (1055–2664)
2060	1424 (837–2238)	3271 (1936–5120)	2217 (1311–3487)
With 2% annual cost inflation			
2020	189 (173–204)	433 (410–457)	294 (269–319)
2025	231 (170–296)	531 (398–672)	360 (265–463)
2030	283 (206–369)	651 (480–838)	441 (322–576)
2035	346 (249–457)	796 (579–1043)	539 (387–712)
2040	422 (297–570)	971 (692–1296)	658 (464–886)
2045	513 (355–713)	1179 (822–1622)	799 (553–1105)
2050	625 (422–879)	1436 (978–1998)	973 (655–1364)
2055	757 (494–1099)	1740 (1142–2503)	1179 (769–1707)
2060	919 (586–1352)	2113 (1355–3094)	1432 (915–2105)
With 4% annual cost inflation			
2020	204 (185–223)	468 (437–501)	317 (287–349)
2025	275 (199–359)	632 (464–815)	429 (311–558)
2030	372 (262–503)	855 (608–1147)	580 (408–783)
2035	502 (342–702)	1154 (793–1602)	782 (532–1088)
2040	677 (441–980)	1555 (1022–2234)	1054 (689–1520)
2045	908 (568–1370)	2087 (1318–3131)	1414 (883–2130)
2050	1223 (731–1905)	2810 (1691–4343)	1904 (1137–2955)
2055	1640 (923–2668)	3768 (2134–6117)	2553 (1439–4144)
2060	2203 (1184–3709)	5062 (2738–8450)	3430 (1854–5772)

95% uncertainty ranges are in parentheses.

**Table 3:** Sensitivity analysis with lower growth rate of ADRDs burden.

Year	Total care purchased in the marketplace (billions of 2020 US$)	Total care purchased in the marketplace plus caregiving time valued according to replacement cost (billions of 2020 US$)	Total care purchased in the marketplace plus caregiving time valued at foregone wage costs (billions of 2020 US$)
Base case with 40% lower burden growth			
2020	195 (178–212)	448 (421–475)	303 (276–331)
2025	243 (177–314)	559 (415–712)	378 (277–489)
2030	303 (218–401)	697 (508–913)	473 (340–624)
2035	378 (267–511)	869 (618–1165)	589 (415–792)
2040	470 (322–651)	1080 (746–1482)	732 (502–1011)
2045	581 (387–831)	1336 (898–1892)	905 (606–1292)
2050	722 (466–1053)	1658 (1083–2397)	1124 (724–1634)
2055	891 (555–1345)	2048 (1283–3073)	1388 (863–2087)
2060	1103 (662–1702)	2535 (1538–3878)	1718 (1039–2646)
With 40% lower burden growth and 2% annual cost inflation			
2020	187 (172–203)	431 (408–454)	292 (267–317)
2025	223 (164–285)	511 (383–646)	347 (255–445)
2030	265 (193–343)	608 (451–781)	412 (301–536)
2035	314 (227–412)	721 (527–937)	488 (353–640)
2040	371 (263–496)	852 (612–1125)	578 (411–771)
2045	437 (306–597)	1004 (710–1356)	680 (477–929)
2050	515 (353–713)	1184 (822–1621)	803 (547–1107)
2055	605 (405–859)	1391 (938–1958)	943 (630–1337)
2060	712 (467–1025)	1637 (1081–2333)	1109 (730–1595)
With 40% lower burden growth and 4% annual cost inflation			
2020	202 (183–222)	465 (435–497)	315 (285–346)
2025	265 (192–346)	609 (447–786)	413 (300–538)
2030	348 (245–469)	799 (570–1071)	542 (382–730)
2035	455 (311–633)	1046 (721–1445)	709 (484–982)
2040	594 (390–856)	1366 (906–1953)	925 (609–1326)
2045	773 (489–1156)	1777 (1132–2636)	1204 (763–1798)
2050	1009 (610–1553)	2318 (1414–3543)	1571 (950–2414)
2055	1311 (755–2107)	3012 (1744–4802)	2041 (1176–3273)
2060	1707 (932–2822)	3923 (2166–6414)	2658 (1466–4389)

95% uncertainty ranges are in parentheses.

**Table 4:** Sensitivity analysis with higher nursing home costs.

Year	Total care purchased in the marketplace (billions of 2020 US$)	Total care purchased in the marketplace plus caregiving time valued according to replacement cost (billions of 2020 US$)	Total care purchased in the marketplace plus caregiving time valued at foregone wage costs (billions of 2020 US$)
Base case with higher nursing home costs			
2020	318 (286–351)	573 (520–628)	428 (377–480)
2025	410 (296–534)	737 (538–959)	551 (395–725)
2030	527 (375–704)	950 (679–1265)	709 (501–956)
2035	678 (473–924)	1220 (852–1665)	911 (630–1252)
2040	869 (587–1222)	1564 (1063–2184)	1168 (783–1645)
2045	1109 (726–1613)	1997 (1316–2895)	1491 (974–2179)
2050	1420 (901–2110)	2557 (1632–3784)	1910 (1199–2850)
2055	1810 (1094–2793)	3259 (1975–5011)	2434 (1465–3773)
2060	2312 (1351–3648)	4162 (2440–6537)	3108 (1820–4916)
With higher nursing home costs and 2% annual cost inflation			
2020	306 (276–336)	551 (503–600)	412 (364–460)
2025	375 (273–485)	675 (496–871)	504 (364–662)
2030	460 (333–602)	828 (601–1085)	618 (442–822)
2035	562 (402–748)	1012 (725–1344)	756 (535–1014)
2040	686 (479–932)	1235 (869–1665)	922 (639–1260)
2045	833 (572–1163)	1500 (1034–2085)	1120 (765–1570)
2050	1014 (681–1432)	1826 (1230–2567)	1364 (905–1934)
2055	1230 (797–1794)	2214 (1438–3213)	1653 (1065–2424)
2060	1493 (948–2206)	2688 (1706–3964)	2007 (1264–2974)
With higher nursing home costs and 4% annual cost inflation			
2020	331 (295–367)	596 (538–657)	445 (389–502)
2025	447 (320–588)	805 (582–1056)	601 (428–798)
2030	604 (422–823)	1088 (764–1477)	813 (564–1114)
2035	816 (553–1145)	1469 (996–2060)	1097 (736–1546)
2040	1099 (714–1603)	1978 (1291–2866)	1477 (954–2153)
2045	1475 (917–2236)	2656 (1664–4005)	1983 (1228–3028)
2050	1986 (1183–3110)	3575 (2141–5573)	2670 (1572–4191)
2055	2663 (1494–4350)	4793 (2697–7804)	3579 (1994–5869)
2060	3578 (1909–6053)	6440 (3456–10810)	4809 (2584–8135)

95% uncertainty ranges are in parentheses.

Assuming an annual cost of care inflation rate of 4%, formal care, formal and informal care (replacement method), and formal and informal care (foregone wages method) costs would rise to $2.2 trillion (95% UR: $1.2– $3.8 trillion), $5.1 trillion (95% UR: $2.7–$8.5 trillion), and $3.4 trillion (95% UR: $1.8–$5.8 trillion), respectively, in 2060. With 4% annual cost of care inflation and 40% lower growth rate of ADRDs burden, these aggregate costs would be $1.7 trillion (95% UR: $932 billion–$2.8 trillion), $3.9 trillion (95% UR: $2.2–$6.4 trillion), and $2.7 trillion (95% UR: $1.5– $4.4 trillion), respectively, in 2060. Projected aggregate costs in all other scenarios would be lower than in the base case.

Considering higher per capita nursing home costs (1.95 times the base case, presented in Supplementary Appendix Table S1), annual aggregate formal care, formal and informal care (replacement method), and formal and informal care (foregone wages method) costs in 2020 were estimated to be $318 billion (95% UR: $286-–$351 billion), $573 billion (95% UR: $520–$628 billion), and $428 billion (95% UR: $377–$480 billion. With a 3% annual inflation rate, these costs were projected to increase to $2.3 trillion (95% UR: $1.4–$3.6 trillion), $4.2 trillion (95% UR: $2.4–$6.5 trillion), and $3.1 trillion (95% UR: $1.8–$4.9 trillion) in 2060. Considering alternative cost of care inflation rates would increase (for 4% inflation) or reduce (for 2% inflation) these projections accordingly.

## Discussion

ADRDs care poses a substantial economic burden in the United States that may more than double in the next two decades. Americans spent $196 billion in direct medical costs for ADRDs in 2020, and as much as another $254 billion in caregiver time was consumed. According to these estimates, formal care for ADRDs therefore comprised almost 5% of all health care spending in the United States and would make up a much larger proportion if patients received more formal paid home-based supports rather than relying on informal caregiving^32^. Estimated costs for ADRDs are larger than for other priority conditions, such as chronic obstructive pulmonary disorder, and comparable to what are considered some of the greatest public health threats, such as all forms of elevated blood glucose levels^33,34^.

With rapid increases in life expectancy and population aging in recent decades, ADRDs have become a major global health threat. If the current status quo continued, the global burden of ADRDs would triple by 2050, resulting in 115.8 million disability-adjusted life years lost every year^30,35^. The global direct and indirect cost (foregone wages method) of dementia care is projected to increase from $1.33 trillion in 2020 to $9.12 trillion in 2050^36^. We found that the United States contributes—and will continue to contribute—substantially to the global economic burden of ADRDs.

Our per capita cost estimates were lower than those of Hurd et al.^6^, on which our methodology was based. The differences are mainly attributable to newer data. Our definition of ADRDs differed slightly from the definition of dementia used in their study. The Affordable Care Act, which was passed in 2010 and has since reduced care costs, coverage gaps, and medical inflation rates, was not captured in the Hurd et al. study, which used 2000–2008 data^27^. A recent study used Medicare claims data from 2006–2015 to estimate an average annual Medicare beneficiary cost of $2,101 for Alzheimer’s disease and $1,870 for general dementia (2015 US$), both of which are lower than the Hurd et al. estimated Medicare cost of $2,752 (2010 US$) per dementia patient^12^. Another 2020 study used Medicare claims data and found that from 2011 to 2016, the 30-day care cost of emergency department visits under Medicare reduced by 8%^11^.

Our future cost projections are within the range of costs estimated by three other studies^7,8,10^. Hurd et al. estimated that if dementia prevalence rates stayed the same as in the ADAMS data, the direct cost, total cost with foregone wage method for indirect cost, and total cost with replacement method for indirect cost would grow to $258.9 billion, $378.7 billion, and $511.4 billion, respectively, by 2040 (2010 US$)^7^. The authors conducted several one-way sensitivity analyses to capture potential changes in prevalence, including those due to comorbidities. In comparison, we conducted a probabilistic uncertainty analysis that captured uncertainties in future disease prevalence growth, population projections, and cost and cost inflation, along with one-way sensitivity analyses. Our corresponding estimates for 2040 were $535 billion, $833 billion, and $1.3 trillion, respectively, with large 95% uncertainty ranges for each (2020 US$). These estimates—adjusted downward for inflation (Consumer Price Index of the U.S. Bureau of Labor Statistics^37^) and expressed in 2010 US$—would be $452 billion, $704 billion, and $1 trillion, respectively. Zissimopoulos et al. projected direct and indirect cost to rise to $1.5 trillion in 2050 (2010 US$)^8^, while the Alzheimer’s Association projected direct cost to rise to $1.1 trillion in 2050 (2015 US$, or $1 trillion in 2010 US$)^10^. In comparison, our 2050 projection of direct cost expressed in 2010 US$ was $739 billion, while total cost, including direct and indirect costs, ranged from $1.1 trillion to $1.7 trillion. These variations can be attributed to differences in underlying data and methodology.

Our findings have important policy implications. ADRDs are projected to impose a fast-growing economic burden in the coming decades, but effective investments in research, prevention, and care could mitigate this^38^. To the extent that people receive the most effective treatments and supports available, prevent or delay onset through changes earlier in life, or get access to new therapies that modify the disease process, the long-term costs of care for ADRDs will decrease as fewer people experience progressive degeneration. Instead, people will age well in place and require less intensive services, despite increasing population age in the coming years. Public-private collaboration in the United States is necessary to mobilize the necessary investment to avert the human and economic tolls of ADRDs^31^.

The health and economic burdens of ADRDs also raise important equity issues. Many risk factors for ADRDs are associated with structural discrimination (e.g., lack of access to education or nutritious foods), so the burdens will be increasingly concentrated among those already in the most challenging financial situations. In the United States, dementia prevalence among those aged 65 years and older is estimated to be 19% and 17% among Black and Hispanic populations, respectively, as compared with 7% among wealthier Whites^39^. In 2020 to 2060, the number of Black, Asian and Pacific Islander, and Hispanic Americans living with ADRDs (65 and older) is estimated to increase 3, 4.7, and 5.4 times, respectively, as compared with a 1.7 times increase among non-Hispanic Whites^40^. Furthermore, almost two-thirds of Alzheimer’s patients in the United States are women, and 10 million women (two-thirds of caregivers) live with or provide care for Alzheimer’s patients^41^.

Investment is required to address these health and economic inequities in the United States. Attention to the ADRDs burden is also important for addressing ageism as a form of discrimination and a domain of health equity. Frequently, conditions that comprise the highest disease burden in childhood or in the working-age population receive the most attention and investment, whereas conditions that affect older adults are often neglected^42^.

Our study has limitations. Most importantly, we did not link HRS respondents with individual Medicare records and approximated Medicare-covered expenses based on Hurd et al.’s estimates^6^. While it is unlikely that Medicare coverage as a share of total cost differed substantially between their and our data, any difference may reduce the accuracy of our estimates. We considered a status quo scenario with a constant growth of disease burden and per capita cost and with no changes in ADRDs treatment protocol or care infrastructure. In addition, we also assumed that the proportions of ADRD patients that receive formal and informal care, as well as the duration of caregiving will remain unchanged in the future. Although we systematically varied inputs in probabilistic uncertainty analysis, they may not fully capture future developments in preventive and therapeutic interventions or care innovations that may change disease burden of caregiving patterns.

Beyond the cost of formal care and the value of informal caregiving, ADRDs may have broader societal and macroeconomic cost implications. Reduction in savings and capital formation due to ADRDs treatment expenditure and lower labor force participation due to mortality and morbidity may reduce long-term economic growth^43^. Due to lack of data, our analysis does not capture these additional costs. Future studies should use modeling techniques that can better account for these other types of societal costs. Finally, to make our estimates comparable to Hurd et al., we used ordinary least squares regression models. Generalized linear models or other techniques may produce more precise estimates for cost variables that have a skewed distribution.

## Methods

### Data

We used data from a nationally representative longitudinal survey of individuals aged 50 and older: the Health and Retirement Survey (HRS). The surveys started in 1992 and cover approximately 20,000 individuals who are interviewed once every two years. The HRS collects data on various aspects of aging, including health and disability, health care access and spending, housing, assets, and employment. A subset of 856 HRS respondents who were age 70 and older in 2000–2002 form the Aging, Demographics, and Memory Study (ADAMS). ADAMS collected in-depth clinical data on cognitive status and dementia of the participants in four waves from 2001 to 2009^6,44^. These data have been used previously to estimate the prevalence of dementia and examine its care and associated costs^6,44–47^. HRS and ADAMS surveys received ethics clearance from the University of Michigan Institutional Review Board^48^. We used publicly available and anonymized secondary data from HRS and ADAMS, and no separate ethics clearance was necessary for our study.

### The probability of ADRDs and cost of care

We used the methodology of Hurd et al. to estimate the probability of ADRDs based on the HRS 2010–2016 data as compared with the HRS 2000–2008 data in their study^6^. First, we used the ADAMS data to estimate an ordered probit model that a respondent had ADRDs, had cognitive impairment but not ADRDs, or had neither. The covariates of this regression included indicators that were also available in the full HRS data: age, sex, schooling attainment, activities of daily living (such as eating and bathing) limitations, instrumental activities of daily living (such as preparing meals or managing money) limitations, and scores on cognitive tests such as identification of the current date, backward counting from 20, word naming, identification of the current U.S. vice president, and immediate word recall. The estimated coefficients of this regression were used to predict the probability of ADRDs for all respondents in the HRS.

Next, we estimated the following regression model for each HRS respondent:

1 $$\cos\! {t}_{{iy}}=\alpha +{\beta }_{1}P{\left({ADRDs}\right)}_{{iy}}+{\beta }_{2}{X}_{{iy}}+{u}_{{iy}}$$

where $\cos\! {t}_{{iy}}$ denotes the cost of medical care for individual *i* in year *y* (2010–2016). Following Hurd et al., we included only individuals aged 70 years and above in our analysis^6^. The estimated probability of dementia, $P{\left({ADRDs}\right)}_{{iy}}$, is from the previous regression, and *X* includes respondent age, sex, household income, categorical variables for education and number of children of the respondent, and a set of binary variables for White or Hispanic and if the respondent has ever had a stroke, lung disease, diabetes, heart disease, hypertension, cancer, psychological conditions, and arthritis. Standard errors of the estimates were obtained through 2000 bootstrap simulations of each model. We report the estimated coefficient ${\beta }_{1}\,$(along with 95% confidence interval [CI] obtained from bootstrapping), which measures the per patient cost of care attributable to ADRDs. Analysis was conducted separately for each $\cos\! {t}_{{iy}}$ category (e.g., formal and informal cost), described in the following.

### Formal cost of care purchased in the market

Formal cost includes out-of-pocket (OOP) spending and costs covered by Medicare and other sources for facility care, formal home care, and nursing home care. OOP costs include expenditure on nursing home stays, hospital stays, doctor visits, dental visits, outpatient surgery, home health care, other special services, prescription drugs, and dental services. We used self-reported HRS data on nights spent at nursing homes and combined them with Genworth’s 2019 data on nightly rates to estimate total nursing home costs paid OOP and through Medicare and other sources^6,49^. Formal home health care includes services provided by an agency and home health aides directly hired by the patient when an individual has difficulty with an activity (basic activities such as bathing or eating) or instrumental activity (more complex activities such as managing finances or using public transportation) of daily living. We used HRS data on hours of home care combined with Genworth unit cost data to estimate the total cost of home care^49^.

Hurd et al.^6^ undertook to link HRS (2000–2008 waves) respondents with their Medicare records and were successful in approximately 70% of the cases. To circumvent the complexity and likely incompleteness of the linking process in the 2010–2016 waves of HRS, we assumed the same coverage rates of Medicare as estimated in the Hurd et al. study. We assumed that Medicare covered 13.3% of total home health care and 8.2% of nursing home care cost. An estimated 57% of Medicare expenses came from nursing homes and home health care, and we used this ratio to extrapolate and project total Medicare costs^6^.

### Informal cost of care, or the caregiver cost

When a person unaffiliated with an agency, such as a family member, provides informal care, there is also a cost. We used two approaches to estimate the informal cost of caregiving: replacement cost and foregone wages. The replacement cost method allowed us to estimate the cost of replacing an informal caregiver with a professional caregiver for the same number of hours. We used Genworth 2019 data for the cost of in-home caregiving for ADRDs. We combined these data with the hours spent by the informal caregiver in the 2010–2016 HRS data to obtain an estimate of professional caregiver replacement cost.

The foregone wage approach estimated the opportunity cost of helping an individual with ADRDs, where the alternative is earning a market wage. We calculated opportunity cost as hours spent by a caregiver helping an ADRD patient multiplied by the market wage that the caregiver could have earned. We obtained wages from the Current Population Survey separately by caregiver demographic characteristics—age, sex, and level of education—and adjusted to 2020 US$ using the consumer price index of the U.S. Bureau of Labor Statistics^50^.

Caregiving time data were available from HRS 2010–2016. For each respondent who received help from a nonprofessional individual, we extracted the number of hours per year of help received. The HRS did not collect hours for caregivers who helped less than once per week, and we imputed values for these observations in each year based on reported caregiver characteristics: sex, relationship to respondent, and number of days per week/month of care. We imputed missing data for caregivers’ demographic traits, education, hours, and wages following a systematic process similar to that of Hurd et al.^6^. The Supplementary Appendix provides additional details of informal cost calculations.

### Future projections

We simulated future cost of ADRDs care by combining estimates for per patient cost as discussed previously with future projections of ADRDs prevalence and medical cost inflation. We obtained 2019 data on ADRDs prevalence from the Global Burden of Diseases (GBD) study of the Institute for Health Metrics and Evaluation, which included all diseases within ADRDs except vascular dementia^6,51^. We estimated the relative prevalence of vascular dementia from the ADAMS data and adjusted the GBD estimates upward^30^. We calculated the average annual growth rate of ADRDs prevalence in the United States during 2010–2019 from the GBD data and assumed that the ADRDs burden will grow at the same rate into the future. The projected future prevalence rate was then combined with population data from the U.S. Census Bureau (2020) and the World Population Prospects (medium variant) of the United Nations to project the number of ADRDs patients from 2020 to 2060^1,52^. We assumed that the formal and informal per patient cost of ADRDs care will grow at an annual rate of 3%, which was the average annual medical care inflation rate in the United States during 2010–2020^53^. The medical inflation rate in the United States is typically higher than the overall annual inflation rate (targeted by the Federal Reserve Bank at 2%)^53^.

We quantified uncertainty surrounding these parameters through a systematic uncertainty analysis. We varied the annual growth rate of ADRDs prevalence, health care inflation rate, population projections within a prespecified range of 75%–125% of the initial value, and the estimated formal and informal cost of care in 2016 within a 95% confidence interval. We drew 10,000 random samples from the joint uniform distribution of these parameters and computed the projected quinquennial future cost of care from 2020 to 2060. We report the mean values with 95% uncertainty ranges from these 10,000 simulations by year.

### Sensitivity analysis

In addition to systematically capturing uncertainty in the parameter values, we conducted several one-way sensitivity analyses by varying the parameters of the model. First, medical expenditure data tend to be right skewed, with some patients having substantially larger (outlier) expenses as compared with the rest. For example, fewer than 10% of HRS respondents in our data have non-zero values for some indicators such as out-of-pocket and total nursing home expenditure. Linear regression may produce imprecise estimates from such skewed data. While the primary purpose of our study is to provide updated estimates that are comparable to Hurd et al. who used similar linear regression models on older HRS data, we also considered the subsample of HRS respondents with non-zero values. We repeated our analysis – for each expenditure variable – only among those who reported positive values. We report these estimates only in per capita terms for 2016, and do not present aggregate estimates or future projections.

Second, recent research has attributed about 40% of all dementia cases to modifiable risk factors such as lack of physical activity, smoking, excessive alcohol consumption, and exposure to air pollution^38,54^. New and future drugs for slowing dementia-related cognitive decline may also reduce the future burden of ADRDs. We considered a scenario in which the base case annual growth rate of ADRDs is reduced by 40%. Third, we considered two additional scenarios with 2% and 4% annual inflation rates of cost of care (instead of 3% as in the base case). We also considered two more scenarios by combining the lower growth rate of ADRDs burden and variations in inflation rates.

Finally, previous research suggests that the HRS may underreport the duration of time spent by respondents in skilled nursing facilities^6,55^. In Hurd et al.’s study, HRS 2008 respondents aged 65 years and above reported spending 9.6 nights on average in a nursing home during the year preceding the survey, which was lower than the Centers for Medicare & Medicaid Services (CMS) based 2007 estimate of 13.8 nights spent in nursing homes^6^. Hurd et al. did not adjust their estimates for this discrepancy, presumably because the proportion of HRS respondents living in nursing homes was similar to that in other sources of data such as the 2000 U.S. Census.

Following Hurd et al.^6^, we compared the reported average duration of nursing home stays in the HRS with data from CMS^56^. Older adults in our HRS 2010 data reported spending 6.4 nights in nursing homes on average per year, which was almost half of comparable estimates from CMS data^56,57^. To account for this difference, we considered an additional scenario where per capita out-of-pocket, Medicare-covered, and total nursing home expenditure were adjusted upwards by a factor of 1.95. The Supplementary Appendix further discusses comparisons between HRS and other data sources data and the rationale for this upward adjustment.

### Supplementary information

Supplementary Information

## Data Availability

The Health and Retirement Study (HRS) data are publicly available from the HRS website https://hrs.isr.umich.edu/.

## Code availability

Code is available from the corresponding author on reasonable request.
