# Supplementary material for: Cost of care for Alzheimer’s disease and related dementias in the United States: 2016 to 2060
Source: NPJ Aging. 2024 Feb 8;10(1):13. doi: 10.1038/s41514-024-00136-6 (PMC10853249; doi:10.1038/s41514-024-00136-6)
Supplement: Supplementary file 1 — Supplementary Information [file 41514_2024_136_MOESM1_ESM.pdf]

Supplementary Information

Supplementary Methods

Formal cost: nursing home care

We use the Genworth Long Term Cost of Care Study[1] for state-level data on nightly private nursing home and hourly home health aide rates combined with the 2010 United States census data made available from the 2019 IPUMS National Historical Geographic Information System[2] to calculate population-weighted region-level data.

Following the methodology used in Hurd et al. (2013)[3], we used the self-reported metric of number of nights spent in a nursing home over the last two years reported in the Health and Retirement Survey (HRS) data and divided the number in half to get the annual number of nursing home nights for that respondent. Total average annual nursing home spending over this period was then computed by multiplying the number of nights in a nursing home with an estimate of the daily rate in the private market for respondents reporting private health insurance. Medicaid rates were used for respondents reporting Medicaid insurance.

As Hurd et al. (2013)[3] and Lima et al. (2018)[4] have noted, HRS may underestimate the time spent by respondents in nursing homes. Similar to the analysis of Hurd et al. (2013)[3], we compared the nursing home occupancy rates and estimated number of nights spent by HRS 2010-2016 respondents to estimates from the 2010 U.S. Census and Centers for Medicare & Medicaid Services data on nursing homes. According to the 2010 Census data, 3.1% of the population aged 65 and older lived in a skilled nursing facility.[5] In comparison, 1.5% of the population aged 65 or older and 3.9% of population aged 70 years or older in the 2010 wave of the HRS sample reported living in nursing homes.

When comparing by age groups, in the 2010 Census, 0.9%, 3.2%, 10.4%, and 24.7% of those 65 to 74 years old, 75 to 84 years old, 85 to 94 years old, and 95 years and older, respectively, lived in nursing homes. In the 2010 wave of the HRS, 0.4%, 1.5%, 5.3%, and 11.9% of those 65 to 74 years old, 75 to 84 years old, 85 to 94 years old, and 95 years and older, respectively, lived in nursing homes.

In 2010, the number of certified beds per 1,000 people aged 65 and older was 41.2 and the occupancy rate was 83.1%,[6] indicating that 34.2 beds per 1,000 were occupied on average per day (or 0.0342 per person). Over a year, those 65 and older spent 12.5 days (0.0342×365 days) on average in a nursing home. In the 2010 wave of HRS, respondents aged 65 and older spent on average 6.4 nights in a nursing home during the previous year (12.7 days in the previous two years). In sensitivity analysis, we considered alternative nursing home costs that accounted for this difference (adjusted upward by a factor of 1.95). Supplementary Table 1 presents the alternative estimates of per patient care costs, which are used in our future projections of aggregate costs.

Supplementary Table 1: Per patient annual cost of ADRDs formal care in the United States (2020 US$)

	Base case estimates	Adjusted estimates (higher nursing home cost)	Estimates for the subsample of non-zero costs
Out-of-pocket:			
Home health care	185	185	239
Nursing home	7,203	14,046	18,298
Total (A)	7,943	14,786	9,945
Medicare spending:			
Home health care agencies	964	964	4,096
Nursing home	1,505	2,935	2,369
Total (B)	4,365	5,795	11,430
Formal home care:			
Total	7,220	7,220	30,675
Less: Medicare and OOP	1,149	1,149	4,335
Net total (C)	6,071	6,071	26,340
Nursing home care:			
Total	18,406	35,892	28,974
Less: Medicare and OOP	8,708	16,981	20,667
Net total (D)	9,698	18,911	8,307
Total care purchased in the marketplace (A+B+C+D)	28,078	45,563	56,022
Informal care: replacement cost (E)	36,667	36,667	36,667
Total care purchased in the marketplace plus caregiving time valued according to replacement cost (A+B+C+D+E)	64,745	82,230	92,689
Informal care: Foregone wage cost (F)	15,792	15,792	15,792
Total care purchased in the marketplace plus caregiving time valued at foregone wage cost (A+B+C+D+F)	43,869	61,355	71,813

Note: Adjusted estimates in the second column represent base case point estimates (manuscript Table 1), of which out-of-pocket, Medicare spending, and total spending for nursing home care were adjusted upward by a factor of 1.95. Non-zero cost estimates presented in the third column exclude individuals with zero costs, separately for each cost category. These estimates are from the base case, i.e., nursing home costs are not adjusted upwards. OOP = out of pocket.

Informal cost of care

Informal care is care provided by a person not affiliated with an agency. We use two approaches to estimate the informal cost of caregiving: replacement cost and foregone wages.

The replacement cost method allows us to estimate the cost of replacing an informal caregiver—usually a family member—with a professional caregiver for the same number of hours.

The foregone wage approach estimates the opportunity cost of being a helper to an individual with ADRDs where the alternative is earning a market wage. The foregone wage method calculates the cost as hours spent by individual z helping individual i multiplied by the market wage that individual z could have earned.

Caregiver hours

We use data on caregiver hours from HRS 2010–2016. We calculate the total number of caregiver hours per respondent; some HRS respondents receive help from multiple individuals, and some individuals help multiple respondents. We focus only on individuals, and drop from our data institutions, organizations, and professional helpers. For each respondent who received help, we extracted the number of hours per year.

Hours spent helping for caregivers who helped less than once per week were not collected, and values for these observations were imputed in each year based on reported caregiver characteristics: sex, relationship to respondent, and number of days per week or month of care, in line with Hurd et al. (2013). For missing values following the imputation, we used linear interpolation to assign yearly hours to individuals who had reported data only in some survey years. Hurd et al. also use place of residence for the imputation. However, in our data residence state or city were hard to come by and only existed for a minority of observations at the household level of the respondent (that is, not the helper). Where no data on hours spent on caregiving (measured in days per week, days per month, or every day in a month) were available, imputation was not possible. One of these three variables is needed for the imputation.

The maximum number of hours per day dedicated to helping in our data day is 24. However, a limit of 16 hours per day was imposed on caregivers, to allow for sleep, similar to Hurd et al. (2013). The hours are reported as hours per day, and number of days are reported as either

- Days per week,
- Days per month, or
- Every day (reported monthly).

We calculate the yearly number of hours:

$$Helper\ hours_{yi} = (hours_{di} * \#days_{mi}) * 12$$

Or

$$Helper\ hours_{yi} = (hours_{di} * \#days_{wi}) * 52.1429$$

Or

$$Helper\ hours_{yi} = (hours_{di} * 30.42) * 12$$

The last equation applies when respondents report helping every day.

The total number of helper hours for individual i in year y is the number of days per month m, multiplied by the number of hours per day d that individual i receives help, and finally multiplied by 12 months.

Supplementary Table 2: Hourly wages from CPS

Hourly wages by age (CPS 2017)				
	**Mean**	**Standard deviation**	**Min.**	**Max.**
**Age 18–34**				
Hourly wage	11.54	4.48	3.87	28.20
in 2020 dollars	12.19	4.73	4.09	29.78
Adjusted by LFPR	9.14	4.59	1.84	26.42
**Age 35–44**				
Hourly wage	20.19	9.58	6.57	52.20
in 2020 dollars	21.32	10.11	6.94	55.12
Adjusted by LFPR	17.42	10.17	3.51	52.48
**Age 45–54**				
Hourly wage	22.07	10.81	7.49	58.51
in 2020 dollars	23.30	11.42	7.91	61.79
Adjusted by LFPR	18.73	11.32	4.13	57.70
**Age 55–64**				
Hourly wage	22.97	11.81	6.93	57.86
in 2020 dollars	24.26	12.48	7.31	61.10
Adjusted by LFPR	16.12	10.81	2.81	49.40
**Age 75+**				
Hourly wage	14.25	6.62	7.48	35.12
in 2020 dollars	15.05	6.99	7.90	37.09
Adjusted by LFPR	1.43	1.47	0.22	6.21
**Total**				
Hourly wage	18.24	10.22	3.87	58.51
in 2020 dollars	19.26	10.79	4.09	61.79
Adjusted by LFPR	11.08	10.96	0.22	57.70

Note: Wage data come from the CPS and are from 2017. Data are first adjusted to 2020 dollars using the CPI and then by the labor force participation rate (LFPR) in each demographic group (age, education, and sex).

Supplementary Table 3: Additional breakdowns of hourly wages

Hourly wages by age (CPS 2017)					
Age	*Education*	*Sex*	*Hourly wage*	*In 2020 dollars*	*Adjusted by LFPR*
18–34	Less than high school	Men	8.2	8.6	5.3
		Women	3.9	4.1	1.8
	High school graduate	Men	14.2	15.0	12.3
		Women	7.8	8.3	5.5
	Some college	Men	14.7	15.5	11.7
		Women	10.3	10.8	7.6
	College graduate	Men	28.2	29.8	26.4
		Women	19.8	20.9	17.4
35–44	Less than high school	Men	15.2	16.1	13.4
		Women	6.6	6.9	3.5
	High school graduate	Men	24.4	25.8	22.5
		Women	12.3	13.0	8.7
	Some college	Men	30.4	32.1	29.0
		Women	16.6	17.5	13.6
	College graduate	Men	52.2	55.1	52.5
		Women	32.3	34.1	28.4
45–54	Less than high school	Men	15.5	16.4	12.0
		Women	7.5	7.9	4.1
	High school graduate	Men	23.7	25.1	20.7
		Women	13.5	14.3	9.6
	Some college	Men	31.6	33.3	28.9
		Women	18.5	19.5	14.9
	College graduate	Men	58.5	61.8	57.7
		Women	34.2	36.1	30.5
55–64	Less than high school	Men	13.8	14.6	8.3
		Women	6.9	7.3	2.8
	High school graduate	Men	24.9	26.3	17.6
		Women	13.7	14.5	7.9
	Some college	Men	28.7	30.3	20.7
		Women	17.6	18.6	11.3
	College graduate	Men	57.9	61.1	49.4
		Women	32.8	34.6	24.2
65–74	Less than high school	Men	12.8	13.5	2.9
		Women	7.2	7.6	0.9
	High school graduate	Men	18.2	19.2	4.8
		Women	11.3	11.9	2.3
	Some college	Men	25.0	26.4	8.3
		Women	15.3	16.1	3.9
	College graduate	Men	44.8	47.4	20.2
		Women	25.3	26.7	8.3

75+	Less than high school	Men	11.8	12.5	1.0
		Women	7.5	7.9	0.2
	High school graduate	Men	16.0	16.9	1.7
		Women	10.4	11.0	0.6
	Some college	Men	19.9	21.0	3.0
		Women	13.8	14.6	1.2
	College graduate	Men	35.1	37.1	6.2
		Women	20.7	21.8	2.6

Note: Wage data are from the CPS and are from 2017. Data are adjusted first to 2020 dollars using the CPI, and then by the labor force participation rate (LFPR) in each demographic group (age, education, and sex).

Foregone wages: wage imputations

Wage imputations were made by matching the average wage in the Current Population Survey 2017 (CPS) among workers in the same demographic group (based on age, sex, and education), as presented in Supplementary Tables 2 and 3. Hourly wage rates were adjusted to 2020 dollars using the Consumer Price Index (CPI). In line with Hurd et al., we multiply the average wage by the labor force participation rate in the same demographic group to account for caregivers who might not work. The labor force participation rate by age ranges from approximately 72% in the youngest age group to 8% among those aged 75 and over.

Demographic characteristics

We observe caregiver age, sex, and education in the HRS data, and we use these characteristics to assign helpers a market wage. We divide available education data into four groups: less than high school, high school graduate or GED, some college, and college graduate.

We also merge education data from the HRS Tracker file, which yields a few thousand additional matches across the years. The education groups are slightly different in this version of the data. We match the available categories in the tracker data to our preferred groups in the following way (Supplementary Table 4):

Supplementary Table 4: Education categories

Education categories	Tracker file data
Less than high school	No degree
High school graduate	High school or GED
Some college	Two-year college degree
College graduate	Four-year degree or higher
Coded as missing	Unknown/some college

If education data are available in multiple data sets, we set education equal to the highest obtained degree.

Age is divided into the following age groups, 18–34, 35–44, 45–54, 55–64, 65–74, and 75+.

Imputations

For any missing observation of age, sex, or education, we attempt cross-matching across survey waves. If values are still missing, we impute the demographic characteristics of respondents as follows:

When only education is missing, we match the education distribution of the caregivers with nonmissing education conditional on age and sex of caregivers with nonmissing values.

For a third of the caregivers, sex was missing, and we assigned a value corresponding to their relationship to the respondent. Sister or daughter would be assigned to female, father or uncle to male, and so on. Finally, any remaining missing observations were assumed to be female, in line with Hurd et al. (2013).

We use the same approach to age imputations as Hurd et al. (2013) and assume that one generation corresponds to 25 years (Supplementary Table 5). Helper relationships include in-laws, other children, and other individuals or relatives. Age has not been imputed when the helper relationship was categorized in the HRS as "Unknown" or "Not a sibling."

If age and education are *both* still missing after this, observations are assigned the median age and education from the survey in year 2000: 57 years old and high school graduate.

Supplementary Table 5: Age imputations for helpers in HRS

Relationship to respondent	Age imputation based on respondent age
Grandchild	-50 years
Child, stepchild, daughter- and son-in-law, other child, other respondent, or other relative	-25 years
Spouse, ex-spouse, late spouse, sibling, brother- and sister-in-law	Same age
Parent or in-laws	+25 years

Supplementary References

1 Genworth. Cost of Long Term Care by State. 2022. https://www.genworth.com/aging-and-you/finances/cost-of-care.html (accessed April 22, 2022).

2 IPUMS. National Historical Geographic Information System (NHGIS). 2019. https://www.nhgis.org/ (accessed Oct 3, 2023).

3 Hurd MD, Martorell P, Delavande A, Mullen KJ, Langa KM. Monetary Costs of Dementia in the United States. *N Engl J Med* 2013; **368**: 1326–34.

4 Lima JC, Ogarek J, Mor V. Untapped Potential – Using the HRS-Medicare Linked Files to Study the Changing Nursing Home Population. *Med Care* 2018; **56**: 216–9.

5 US Census Bureau. The Older Population: 2010. US Department of Commerce, 2011.

6 Centers for Medicare & Medicaid Services. Nursing Home Data Compendium 2015. Washington DC, 2015.
